# Interpretation bias modification to reduce body dissatisfaction – a randomized controlled pilot study in women with elevated weight and shape concerns

**DOI:** 10.1186/s40337-020-00305-4

**Published:** 2020-07-02

**Authors:** Silvia Bradatsch, Marlene Dorit Vahl, Rachel Potterton, Gemma Gordon, Ulrike Schmidt, Timo Brockmeyer

**Affiliations:** 1 Department of Clinical Psychology and Psychotherapy, Institute of Psychology, University of Goettingen, Goettingen, Germany; 2 Section of Eating Disorders, Department of Psychological Medicine, Institute of Psychiatry, Psychology and Neuroscience, King’s College London, London, UK

**Keywords:** Eating disorders, Information processing, Negative interpretation bias, Cognitive bias modification, Scrambled Sentences Task, Body dissatisfaction, Body image

## Abstract

**Background:**

Recent research has identified several cognitive biases in patients with eating disorders, such as a tendency to interpret ambiguous information about one’s own body in a negative way. The so-called “negative interpretation bias” is considered to be a key factor in maintaining maladaptive cognitions and behaviors in eating disorders. Studies on modification of the negative interpretation bias in eating disorders have yielded mixed results. This randomized controlled pilot study examined whether a specially adapted, computerized version of the Scrambled Sentences Task modifies negative interpretation bias in women with elevated body dissatisfaction.

**Methods:**

The sample consisted of 40 normal-weight women with elevated body dissatisfaction, randomly assigned either to an intervention or a no-intervention control group (each *n* = 20). The intervention group received six sessions (within two weeks) of a newly-developed interpretation bias modification training that involved unscrambling positively valenced, body image-related sentences. The control group received no intervention. In both groups, body image-related negative interpretation bias (main outcome), trait body dissatisfaction and thin-ideal cue reactivity were assessed at baseline and two weeks later. Additionally, in the intervention condition, the trajectory of expected reductions in the thin-ideal internalization was measured during each training session.

**Results:**

In both conditions, body image-related negative interpretation bias and trait body dissatisfaction decreased significantly from pre- to post-assessment; however, a specific effect imparted by the interpretation bias modification training was not found. Groups did not differ in thin-ideal cue reactivity. In the intervention group, thin-ideal internalization decreased significantly over the training sessions.

**Conclusions:**

The findings do not support use of body image-related interpretation bias modification in its current form in the treatment of body dissatisfaction. Further research involving different versions of the training and clinical samples is warranted.

## Plain English summary

People with an eating disorder, or at risk of it, often show a so-called negative interpretation bias: they tend to interpret ambiguous information, e.g. concerning their own body, in a negative way. Recent research has begun to study interventions that try to change this bias, as it is linked with eating disorder symptoms like being unhappy about your body and is thought to keep the disorder going. The present study tested a newly developed intervention in 40 women who had normal weight but were unhappy about their body: 20 had the training, and 20 did not. The 6-session training involved unscrambling positive, body image-related sentences, e.g. “My body is quite beautiful/attractive”. We found a decrease in body image-related negative interpretation bias and body dissatisfaction from before to after the intervention. However, these improvements were not specific to the training as the women in both groups showed comparable changes. Hence, in its current form, the training cannot be recommended as an intervention for body dissatisfaction.

## Background

In the development and maintenance of eating disorders, cognitive schemata take a key role as they are assumed to affect thoughts and behavior in a maladaptive manner and lead to excessive preoccupation with one’s own body and eating behavior [1–5]. These schemata foster biased information processing [2, 6, 7], such as the tendency to interpret ambiguous information in a negative manner (e.g. [8]). In terms of eating disorders, this may concern information on the person’s own body, weight or shape [9]. As these cognitive biases are rather implicit in nature (e.g. [2]), it may be difficult to address and modify them when drawing on conventional cognitive-behavioral psychotherapeutic techniques. Thus, it may be beneficial to develop specific interventions that take into account the nature of these cognitive biases, which was the aim of the current study.

Negative interpretation bias is commonly assessed using fictional ambiguous scenarios (e.g. two friends giggling behind oneself), where participants are either required to spontaneously generate an interpretation (sentence completion task), or to rate the likelihood of a *given* interpretation [10, 11]. However, these measurements are rather explicit in nature and may thus be susceptible to demand characteristics, selection and response bias [12], as well as social desirability [13]. Implicit measures for negative interpretation bias serve as a helpful addition since they are less affected by cognitive control and enable capturing of less deliberate, spontaneous cognitive processes [13, 14].

The so-called *Scrambled Sentences Task* (SST) is such an implicit measure that was originally developed to assess negative interpretation bias in patients with depression [15]. More recently, the SST has been adapted for eating disorders to examine whether patients tend to interpret ambiguous body image-related information in a negative way [9]. In this computerized version, participants have to rearrange self-referential scrambled sentences containing six words (e.g. “AM NOT I REALLY TOO FAT”) by using only five of these words. Depending on the words chosen, this can either result in a positive (“I AM NOT TOO FAT”) or a negative statement (“I AM REALLY TOO FAT”). It is crucial to the task that it also comprises neutral distractor items [16], and that participants operate under cognitive load and time pressure [15]. Using the SST in a previous study, we found that patients with Anorexia Nervosa show a stronger body image-related negative interpretation bias than healthy controls [9]. Furthermore, body image-related negative interpretation bias was positively associated with eating disorder symptom severity and body dissatisfaction in both patients and healthy women [9]. More recently, interpretation bias has also been investigated as a potential mechanism in the development of eating disorders: After a training that reinforced negative interpretations, healthy women showed increased body dissatisfaction and reduced appearance-related self-esteem [17]. This is noteworthy as both high body dissatisfaction and low appearance-related self-esteem represent considerable risk factors for eating disorder development. Together these findings suggest that reducing body image-related negative interpretation bias could be a promising avenue in the prevention and treatment of eating disorders.

Cognitive bias modification (CBM) training provides an easily accessible tool for this purpose. CBM paradigms targeting interpretation bias (CBM-I) foster positive (i.e., unbiased, balanced, or self-serving) interpretations of ambiguous information [18, 19]. Despite mixed results in meta-analyses [20, 21], CBM-I has shown potential to reduce psychopathology [22]. So far, seven studies have examined CBM-I in the domain of body image/ eating disorders (for review see [19]), of which four studies investigated *appearance-*based CBM-I approaches [23–27]. The latter showed significant positive effects on negative interpretation biases as well as corresponding symptomatology, i.e. body dissatisfaction, body image disturbance (in terms of body size overestimation) and eating disorder pathology. Effect sizes were moderate to large (for details see [19]), which is notable given that the studies varied in 1) the examined sample, 2) the number of training sessions, and 3) the nature of the intervention/ task. However, to what degree the training paradigms that were used actually address implicit/ automatic processes may be questioned. In three of these paradigms, an ambiguous scenario is described that is either preceded by a benign connoted word (e.g. “cheerful”) [23, 24], positively resolved by the final word of the scenario [23, 24, 26], or that has to be imagined in a positive way [25]. The fourth paradigm involved categorical (thin vs. fat) judgments of varying body sizes, favoring fatness judgments [27]. As participants answer a comprehension question and/or receive feedback after each trial [23, 24, 26, 27], and positive imagination is particularly instructed [25], these CBM-I interventions can be seen as rather explicit, i.e. addressing deliberate, conscious cognitive processing. In turn, we suggest that it might be worthwhile to also address more automatic/ unconscious, i.e. *implicit*, cognitive processes. Interpretation biases do not only operate consciously but are also involved in early, more automatic stages of stimulus processing, thereby occurring outside of awareness, cognitive control and intention [12]. Hence, the present study aims to test a more implicit CBM-I intervention which was newly developed as an adaptation of the SST [9]. We further chose to use a training paradigm that forces participants to make positive judgments regarding their own body, which is contrary to scenario ambiguity in the aforementioned studies. We consider the latter as a potentially critical point: Ambiguity may trigger a negative interpretation first, making it difficult to switch and acknowledge the final positive scenario outcome. Moreover, CBM-I interventions have been shown to be especially effective when combined with a stress induction [22]. Encountering a stressor activates the cognitive schemata and biased information processing targeted by the intervention. We implemented this by exposing participants to photographs of fashion models meeting the thin-ideal. To assess their specific reaction to these cues, a differentiation between trait and state body dissatisfaction is necessary [28–30]: While most research relies on dissatisfaction as a trans-situational and relatively stable trait, we also measured state body dissatisfaction as a snapshot of the current perception and satisfaction with the own body after exposure to thin-ideal cues [31].

This pilot study tested a novel CBM-I intervention for body image-related negative interpretation bias in an analogue sample of young women with elevated levels of body dissatisfaction. The computer-based training comprised an adapted version of the SST [9], in which participants are forced to build positive statements regarding their own body. We hypothesized that, as compared to the control group, the intervention group will show a stronger pre- to post-assessment reduction of (a) body image-related negative interpretation bias and (b) self-reported trait body dissatisfaction. We further hypothesized that (c) the extent of the reduction in body image-related negative interpretation bias will mediate the effect of group (intervention vs. control) on trait body dissatisfaction. Lastly, we hypothesized that women in the intervention group will show (d) reduced cue reactivity (i.e. a smaller increase in state body dissatisfaction after exposure to thin-ideal cues) compared to the control group at post-measurement, and (e) a linear decrease in self-reported thin-ideal internalization over the course of the six training sessions.

## Methods

### Study design

This study was a non-blinded, single-center, pilot randomized-controlled superiority trial utilizing a parallel group design. Participants were randomly allocated to one of two conditions (CBM-I vs. no-intervention control group). Change in body image-related negative interpretation bias was the primary outcome. Secondary outcomes were changes in self-reported trait body dissatisfaction and thin-ideal cue reactivity. All outcomes were assessed at pre- and post-assessment. The study protocol was approved by the local institutional review board.

### Participants

The total sample consisted of 40 adult women with elevated self-reported body dissatisfaction. Inclusion criteria were: female, age ≥ 18 years, normal weight according to WHO guidelines (Body Mass Index between 18.5 and 24.9 kg/m^2^), a score ≥ 1 *SD* above the mean of the norm sample on the German version of the *Body Shape Questionnaire* (BSQ; [32]) as well as German as mother tongue (due to the rather complex, text-based intervention). Participants were recruited via adverts on the campus of the University of Goettingen and via social media. As we expected a medium effect size for the present study (based on previous studies of CBM and similar interventions, e.g. [22, 33–35]), an a priori power analysis for a mixed ANOVA with 0.05 two-tailed significance level and 80% power revealed a minimum sample size of *N* = 34. To compensate for potential data loss of *α* = 15% due to dropouts or technical errors, a correction factor of 1/1-*α* was applied, resulting in a sample size of *N* = 40 (i.e. *n* = 20 per group). All participants provided written informed consent and received either financial remuneration (€ 30) or course credit for their participation in the study.

### Measures

#### Primary measures

##### Body image-related Scrambled Sentences Task

**Fig. 1:**
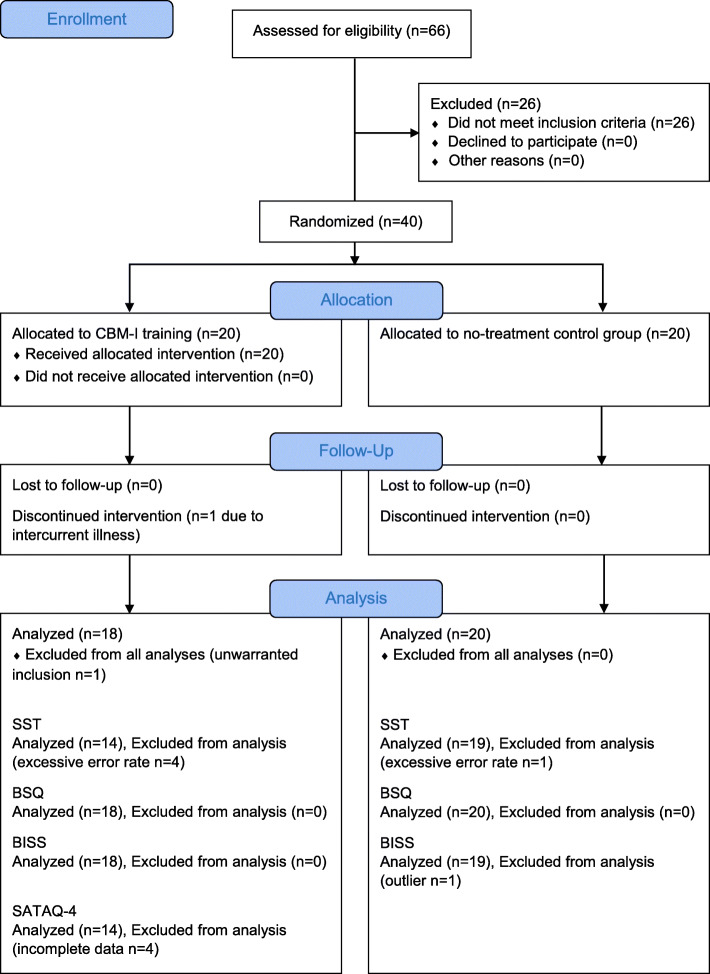
CONSORT flow diagram

Body image-related negative interpretation bias (i.e. a tendency to interpret ambiguous information related to the own body as negative) as measured by the SST served as the primary outcome [9] (original version: [15]). The SST is an established measure for interpretation bias, originally developed for research in depression (e.g. [36, 37]). Here we used an adapted version of the SST that measures body image-related negative interpretation bias [9]. Twenty scrambled, body image-related sentences, each containing six words, were presented one by one on a computer screen (e.g. “BE THIN LIKE HEALTHY TO I”). Within 10 s, participants had to build a grammatically correct sentence by re-arranging five of the six words via mouse clicks. Unscrambling could result in either a positive (“I LIKE TO BE HEALTHY”) or a negative statement (“I LIKE TO BE THIN”). To prevent primacy and recency effects, target words were neither displayed at the beginning or ending of the word string nor next to each other. Also, positive target words were presented before the negative target word in exactly half of the sentences, and vice versa, and target word pairs were matched for word class, word length, and frequency in the German language. Twenty neutral, body-*unrelated* sentences were added as distractor items (e.g. “I REALLY LIKE WRITING LETTERS/ EMAILS”) that serve to conceal the purpose of the measure [16]. The time limit of 10 s aims to prevent ceiling effects [15]. In order to increase cognitive load, participants additionally had to memorize a random, computer-generated 6-digit number during the SST. The number was presented once at the beginning of the task and participants had to reproduce it from memory at the end. Cognitive control and social desirability tendencies thereby decrease [15], facilitating the presentation of implicit cognitive processes as intended. Negative interpretation bias was defined as the proportion of negative target sentences constructed over correctly completed target sentences. Five participants had to be excluded from the SST analysis due to an excessive error rate of more than 65% grammatically incorrect target sentences (cf. [9]), resulting in a sample size of *n* = 14 in the CBM-I group and *n* = 19 in the control group (Fig. 1).

#### Secondary measures

##### Body Shape Questionnaire

Trait body dissatisfaction was assessed by the German version of the BSQ [32].The BSQ is a standardized and widely used questionnaire in the field of body image research. Previous studies demonstrated excellent internal consistency of the BSQ (*α* = .95 and .96 in non-clinical samples) and strong correlations with specific subscales of the *Eating Disorder Inventory* (*Body Dissatisfaction*: *r* = 0.71 and *Drive for Thinness*: *r* = 0.70 in a sample of women with Bulimia Nervosa; [32]). Thirty-four items capture different aspects of body dissatisfaction on a 6-point Likert scale (from 1 = *never* to 6 = *always*), i.e. higher mean scores reflect higher levels of body dissatisfaction.

##### Body Image States Scale

State body dissatisfaction was assessed by using the German version of the *Body Image States Scale* (BISS; [31]) before and after exposure to photographs of fashion models meeting the thin-ideal (for similar procedures of assessing cue reactivity see for instance [38, 39]). The BISS has shown acceptable to excellent reliability (internal consistency in a female sample: *α* = .77–.90 across different contexts), and context sensitivity and construct validity were confirmed [31]. Participants answer six items on how they currently feel about their body on a 9-point Likert scale (for example, from 1 = *extremely unsatisfied with my appearance* to 9 = *extremely satisfied with my appearance*). Hence, lower values indicate higher dissatisfaction with appearance, weight and figure, as well as a more negative evaluation of one’s own attractiveness. This procedure (i.e. presenting the questionnaire before and directly after cue exposure) was chosen in order to activate cognitive schemata related to body image. For each participant, we calculated the difference between the BISS score before and after cue exposure, resulting in a change score that indicated participants’ thin-ideal cue reactivity.

*Thin-ideal cues.* Selection of the pictures was based on the following: First, a set of 50 images displaying thin models was selected from popular magazines and fashion brands from the internet. Criteria for this selection were: (a) pictures should display only one single woman, (b) her whole body (i.e. head to toes) had to be visible and not obscured by baggy clothing (i.e. women in the pictures wore short dresses or skirts/ shorts and a tight top or bra), (c) her physique should reflect the thin-ideal (i.e. she had to be particularly lean), (d) she had to be within 45° of facing front, (e) she had to appear at least 18 years old and (f) not pregnant, and (g) pictures had to be of sufficient quality, and (h) without much distracting content. In a second step of this task preparation procedure, nine young women (i.e. eating disorder researchers; between 25 and 35 years old) were asked to independently rate how much the 50 pictures meet the thin-ideal by using a Likert scale ranging from 0 (*totally not representative*) to 9 (*totally representative*). The raters were told that they should keep in mind that the pictures would be used to trigger body dissatisfaction in young women with elevated weight and shape concerns when they compare themselves to the women displayed in the pictures. As a meta-analysis [40] found a trend towards greater effects for a small number of cues (i.e., 1 to 9) on body dissatisfaction, the 7 pictures with the highest mean rating scores were selected (range: 7.56–8.67; *M* = 8.06; *SD* = 0.34, Cronbach’s *α* = .75 across the 7 pictures). All of the pictures displayed very thin models wearing light clothing and the duration of picture presentation was 2:17 min.

##### Sociocultural Attitudes Towards Appearance Questionnaire-4

In order to get a more fine-grained picture of the trajectory of expected decreases in body dissatisfaction during the intervention, participants in the intervention group additionally completed the subscale *Internalization: Thin/Low Body Fat* of the *Sociocultural Attitudes Towards Appearance Questionnaire-4* (SATAQ-4; [41]) at the end of each training session. The SATAQ-4 contains different measures for body image, self-confidence and disturbances of eating behavior. Previous research has shown good to excellent internal consistency (*α* = .82–.95 in female samples) and construct validity was confirmed [41]. The subscale consists of five items answered on a five-point Likert scale (from 1 = *I definitely disagree* to 5 = *I definitely agree*), with higher values reflecting higher internalization of the female thin-ideal.

### Intervention: CBM-I training

We used a modified version of the SST for the interpretation bias modification training. While the assessment version allowed for either creating a negative or a positive unscrambled statement about the own body, it was solely possible to form positive body image-related statements in the training version. Eighty self-referential and body image-related sentences were generated, each allowing for a minimum of two positive outcomes (e.g. “MY BODY IS QUITE BEAUTIFUL/ MY BODY IS QUITE ATTRACTIVE”). In a pretest, female psychology students (*N* = 10) reviewed these sentences regarding speech quality, fluency in daily use, and potential reactance that may foster unscrambling of exaggerated or inappropriately positive sentences. Based on this, a set of 40 sentences was chosen for the training, which consisted of six sessions (10 min. each) of computerized training within two weeks (for information on the optimal amount of training sessions in CBM see [42]).

Participants in the control group received no intervention but underwent the same assessments as participants in the intervention group, except for the SATAQ-4.

### Procedure

Study adverts on printed posters and social media postings contained a link to a website that provided detailed information about the study, i.e. the study’s background, its course, and potential training benefits with regard to body image and body dissatisfaction. The website also comprised a screening questionnaire, the results of which individually determined study eligibility. Eligible individuals were contacted via e-mail and invited to the laboratory. After providing written informed consent, participants were randomly assigned to either the intervention or the control group. To this end, a computer-based randomization program was used (Research Randomizer; [43]). Randomization was performed in a 1:1 ratio; results were stored in sealed envelopes to ensure allocation concealment.

All participants attended two appointments for computer-based assessment at an interval of two weeks. First, participants performed the SST assessment version [9] as a measure for body image-related negative interpretation bias. They also answered the BISS [31] twice, once before and once after the presentation of seven visual cues that should increase state levels of body dissatisfaction. Afterwards, they answered the BSQ [32] on trait body dissatisfaction. The intervention group received a short, standardized introduction to the CBM-I training and completed the first session in the lab. The following four training sessions were conducted via the internet, within two weeks. The investigator monitored adherence to the training schedule and sent an email reminder if necessary. To assess changes in the degree of internalization of the thin-ideal over the course of training, participants completed the SATAQ-4 [41] after each session. The sixth (final) session of training and completion of the SATAQ-4 [41] took place in the lab again. Following this, both groups completed the SST assessment version and the BISS before and after cue exposure [31], as well as the BSQ [32] again.

### Data analysis

SPSS 25 was used for the statistical analyses. We conducted Welch’s *t*-tests to compare both groups regarding age, BMI, primary and secondary outcomes at pre-assessment. We also calculated Pearson correlations of primary and secondary outcomes at pre-assessment. To analyze pre- to post-measurement changes in both groups, we conducted several 2 × 2 mixed ANOVA; changes during the intervention were investigated using repeated-measures ANOVA. Assumptions of the statistical analyses were checked beforehand, and no relevant deviations had occurred. Statistical significance was defined at the .05 *p*-level, two-tailed; effect sizes display partial *η*^2^, with *η*^2^ = 0.01 corresponding to a small effect, *η*^2^ = 0.09 to a medium effect, and *η*^2^ = 0.25 to a large effect [44].

## Results

### Descriptive statistics

The final sample consisted of *N* = 38 women (per-protocol analysis; intervention group: *n* = 18; control group: *n* = 20) with a mean age of *M* = 23.03 years (*SD* = 4.61, range 19–43 years) and a mean BMI of *M* = 21.99 (*SD* = 1.79, range 18.52–25.08). There were two dropouts, one due to intercurrent illness and another due to an error in the screening process whereby a participant had been included despite her BMI of 28.76, which exceeds the normal range according to the WHO (Fig. 1). Due to an administrative error, two participants were recognized as being slightly overweight (BMI of 24.96 and 25.08, respectively) only after they had already undergone the pre-assessment. Since they were approximately normal weight, and only slightly exceeding the weight criterion was not a strict contraindication to the intervention, we decided to not exclude these participants. Besides, excluding these participants from the analysis did not change the pattern of results. Table 1 summarizes the sample characteristics of the intervention vs. control group and the *t*-statistic referring to pre-measurement comparisons: The groups did not differ in age or BMI, nor in their baseline level of primary (negative interpretation bias) or secondary outcomes (trait body dissatisfaction and thin-ideal cue reactivity). At pre-assessment, stronger body image-related negative interpretation bias was highly correlated with greater trait body dissatisfaction (*r* = .76, *p* < .001) as well as greater state body dissatisfaction before (*r* = −.79, *p* < .001) and after cue exposure (*r* = −.82, *p* < .001; note that higher body dissatisfaction is reflected by lower BISS scores). It was, however, not correlated with thin-ideal cue reactivity, i.e. the BISS change score (*r* = −.19, *p* = .29).

**Table 1:** Sample characteristics

	Intervention group		Control group		
	Pre-assessment	Post-assessment	Pre-assessment	Post- assessment	*t*-statistic pre-assessment comparisons
	*M* (*SD*)	*M* (*SD*)	*M* (*SD*)	*M* (*SD*)	
Age (years) (*n*_i_ = 18; *n*_c_ = 20)	22.61 (2.97)	–	23.40 (5.75)	–	*t*(36) = 0.52; *p* = .61
Body Mass Index (kg/m^2^) (*n*_i_ = 18; *n*_c_ = 20)	22.52 (1.85)	–	21.52 (1.63)	–	*t*(36) = − 1.77; *p* = .09
Body image-related negative interpretation bias					
SST assessment version (*n*_i_ = 14; *n*_c_ = 19)	0.55 (0.22)	0.44 (0.22)	0.60 (0.26)	0.57 (0.31)	*t*(31) = 0.62; *p* = .54
Body dissatisfaction					
Trait					
BSQ (*n*_i_ = 18; *n*_c_ = 20)	3.12 (0.91)	2.85 (0.87)	3.47 (0.85)	3.28 (0.87)	*t*(36) = 1.23; *p* = .23
State					
BISS prior to cue exposure (*n*_i_ = 18; *n*_c_ = 19)	3.27 (1.10)	3.44 (1.09)	2.83 (1.17)	3.31 (1.44)	*t*(35) = − 1.16; *p* = .25
BISS after cue exposure (*n*_i_ = 18; *n*_c_ = 19)	2.97 (1.10)	3.24 (1.25)	2.27 (1.31)	2.85 (1.57)	*t*(35) = − 1.76; *p* = .09
Thin-ideal cue reactivity					
Changes on BISS (from before to after cue exposure) (*n*_i_ = 18; *n*_c_ = 19)	−0.30 (0.53)	− 0.20 (0.41)	−0.56 (0.62)	− 0.46 (0.58)	*t*(35) = − 1.39; *p* = .17

*Note*. i = intervention group; c = control group; SST = Scrambled Sentences Task; BSQ = Body Shape Questionnaire; BISS = Body Image States Scale, before and after presentation of fashion model photographs

### Primary outcome: body image-related negative interpretation bias

In a 2 × 2 mixed ANOVA (intervention group: *n* = 14; control group: *n* = 19), we did not find the expected interaction effect of group and time (pre- vs. post-assessment) on body image-related negative interpretation bias, *F*(1, 31) = 3.88, *p* = .06., *η*^2^ = .11. Contrary to our hypothesis, participants in the intervention group did not show a stronger reduction of interpretation bias after CBM-I training when compared to the control group. Instead, we found a significant main effect of time, *F*(1, 31) = 11.47, *p* = .002, *η*^2^ = .27, indicating that both groups featured a reduction in interpretation bias from pre- to post-assessment. The main effect of group on interpretation bias did not reach statistical significance, *F*(1, 31) = 1.10, *p* = .30, *η*^2^ = .03.

### Secondary outcomes: trait body dissatisfaction and thin-ideal cue reactivity

A 2 × 2 mixed ANOVA (intervention group: *n* = 18; control group: *n* = 20) did not yield the hypothesized interaction effect of group and time on trait body dissatisfaction, *F*(1, 36) = 0.66, *p* = .42, *η*^2^ = .02. In comparison to the control group, participants of the intervention group did not show lower levels of trait body dissatisfaction after CBM-I training. Again, there was a significant main effect of time, *F*(1, 36) = 23.73, *p* < .001, *η*^2^ = .40, indicating that both groups showed lower levels of trait body dissatisfaction at post-assessment than at pre-assessment. The main effect of group on trait body dissatisfaction did not reach statistical significance, *F*(1, 36) = 1.96, *p* = .17, *η*^2^ = .05.

Concerning thin-ideal cue reactivity, we first did a manipulation check whether state body dissatisfaction indeed increased as intended after the presentation of the visual cues. One statistically significant outlier (z-value > 3 SD) was excluded (intervention group: *n* = 18; control group: *n* = 19; see Fig. 1) before we ran two separate 2 × 2 mixed ANOVAs with group as between-subjects factor and time (before vs. after cue exposure) as within-subjects factor, for both pre- and post-assessments. Both times, we found state body dissatisfaction to be increased after cue exposure in both groups, i.e. BISS scores decreased from prior to after cue exposure, *F*_pre_(1, 35) = 20.23, *p* < .001, *η*^2^ = .37; *F*_post_(1, 35) = 15.61, *p* < .001, *η*^2^ = 0.31. Neither the main effects of group, *F*_pre_(1, 35) = 2.30, *p* = .14, *η*^2^ = .06; *F*_post_(1, 35) = 0.36, *p* = .55, *η*^2^ = .01, nor the interaction effects of group and time reached statistical significance, *F*_pre_(1, 35) = 1.93, *p* = .17, *η*^2^ = .05; *F*_post_(1, 35) = 2.29, *p* = .14, *η*^2^ = .06.

Since the manipulation worked as intended, we conducted a 2 × 2 mixed ANOVA with group as between subjects factor, time (pre- vs. post-assessment) as within-subjects factor and thin-ideal cue reactivity (i.e. changes on the BISS from prior to after cue exposure) as dependent variable. The expected interaction effect did not reach statistical significance, *F*(1, 35) = 0.01, *p* = .94, *η*^2^ < .001. In comparison to the control group, participants in the intervention group did not show an attenuated reaction to thin-ideals after CBM-I training. The main effects of group, *F*(1, 35) = 2.76, *p* = .11, *η*^2^ = .07, and time, *F*(1, 35) = 1.25, *p* = .27, *η*^2^ = .03, were also not statistically significant.

#### Mediation analysis

As there was no significant interaction effect of group (intervention vs. control) and time on self-reported trait body dissatisfaction, we refrained from testing the hypothesized mediation model.

### Further analysis: change trajectory of thin-ideal internalization during the intervention

In order to analyze changes in thin-ideal internalization over time in the intervention group, we conducted a repeated-measures ANOVA. Participants were included in this analysis if they had completed the questionnaire six times (once after each training session). This resulted in a sample size of *n* = 14 (Fig. 1). As expected, SATAQ-4 scores decreased over time, *F*(5, 65) = 4.54, *p* = .001, *η*^2^ = .26. The polynomial trend analysis suggested a linear trend as the best fitting model, *F*(1, 13) = 12.54, *p* = .004, *η*^2^ = .49.

## Discussion

The present randomized controlled pilot study tested the efficacy of a novel body image-related interpretation bias modification training in an analogue sample of young women with elevated levels of self-reported body dissatisfaction. Contrary to our hypothesis, participants who received six sessions of this intervention did not show stronger reductions in body image-related negative interpretation bias and trait body dissatisfaction than participants who did not receive an intervention. Instead, participants in both conditions showed reduced interpretation bias and trait body dissatisfaction at post-assessment. Furthermore, women in the intervention group did not show reduced thin-ideal cue reactivity when compared to the control group at post-assessment. Concerning levels of thin-ideal internalization, we found a decrease over the course of the six training sessions in the intervention group, following a linear trend. However, as we did not measure this variable in the control group, we cannot rule out that the same would apply for these participants. Hence, in its current form, the intervention cannot be recommended for the treatment of body dissatisfaction.

There are several possible explanations for these unexpected results. First, our sample size may have been too small to detect an effect of the intervention on body image-related negative interpretation bias. As some participants had to be excluded from the analysis, the sample size was slightly smaller than the a priori power analysis suggested. Indeed, a medium effect size was observed, but due to the effect being not statistically significant, we refrained from post-hoc testing at this point and recommend larger samples for further studies. Secondly, the training intensity may have been too low, since it comprised only six 10-min sessions within two weeks. In contrast, earlier studies achieved positive results, at least on negative interpretation bias, when using CBM-I of comparable or even shorter length [23–27, 45–47]. When CBM-I consisted of a single session, however, post-assessment took place directly after training and longer-term efficacy remained unclear [25, 26, 46], or the effects were not entirely maintained at follow-up [47]. Thirdly, participants ran four training sessions online (i.e. at a time and place of their convenience on personal devices). This implies less control over adequate performance of the intervention [48], e.g. participants may have been distracted or inattentive. On the other hand, this kind of intervention is meant to be easily accessible. Potentially, an improved version could include feedback regarding participants’ performance in order to increase their compliance. Fourthly, our CBM-I training differed in several parameters from previously used, more effective CBM-I paradigms for appearance and body image concerns, which are explained below.

Our CBM-I training used positive sentences directly referring to the participant’s own body (e.g. “My body is quite beautiful/attractive”), instead of scenarios that depict an ambiguous situation where body-related content is relevant (e.g. “You see your reflection in a window”, [23], p. 121). This narrowly defined task content may have lacked a potentially crucial feature of body image/ dissatisfaction, namely social evaluation (i.e., by others). Some studies support the idea that women’s evaluation of their own bodies is affected by their presumption of how others judge their appearance [e.g. 33]. Indeed, the study by Summers and Cougle [23] that explicitly targeted social evaluation- *and* appearance-related interpretation bias yielded the strongest effect sizes compared to other appearance-based approaches [25–27]. Some of the latter, however, indirectly contained social aspects as well, as the scenarios also involved other people (e.g. going for a hike with a friend or having exercised at a health club; [25, 26]). Thus, the intervention’s efficacy appears to vary with the degree of social evaluation being involved. Moreover, individuals with elevated body dissatisfaction might be less likely to self-identify with sentences that describe their body directly – and might be more likely to identify with sentences that focus on the social evaluation of their body. Our CBM-I training might thus have been more effective if we included social evaluation-focused sentences (e.g. “Others really appreciate/like my body.”).

Our CBM-I paradigm further differed from others in terms of its conceptualization as a rather implicit training modality. We thereby aimed to modify automatic cognitive processes in a more subtle way than had previously been done. However, in view of the results, our CBM-I training appears less promising and considerably less effective than more explicit interventions. It is also not entirely clear to which degree implicit measures like the SST are able to capture implicit processes [49]. Thus, it is uncertain to what extent it can *modify* implicit processes. Disregarding this general limitation, we attempted to design a task that operates as implicitly as possible: By increasing cognitive load during the task (i.e. memorizing a random 6-digit number) and setting a time limit of 10 s per sentence, we attempted to decrease cognitive control [15] and thereby highlight implicit processes. However, as participants had to memorize the number at the beginning of the task and reproduce it at the very end, cognitive load during the SST itself may not have been sufficiently high. Moreover, correct recall was neither incentivized nor rewarded and participants only received brief feedback on their accuracy. Prospectively, requesting participants to reproduce the number several times (e.g. after each 20 of the 80 trials) and offering a monetary incentive for correct recall may encourage stronger engagement with this cognitive task, thereby increasing the likelihood that it works as intended.

Although we did not find a specific effect of the CBM-I intervention, a decrease of interpretation bias and body dissatisfaction over time occurred in both groups. It is unlikely that these changes are simply due to random improvements since effect sizes were large. A possible explanation may be that study participation itself facilitated participants becoming more aware of their body dissatisfaction – which may in turn have encouraged them to take independent action to improve body dissatisfaction. However, this would imply that (self-) improved body dissatisfaction is interwoven with improvements in negative interpretation bias. It has not yet been investigated to what extent this causal relationship may exist. A more reasonable explanation for these results is regression to the mean. It describes the statistical effect that high/ extreme pretest scores trend towards the population mean at posttest. Furthermore, our no-intervention control group differed from previous, similar studies which used a placebo control group [23–27]. In future studies, it may be beneficial to include both of these, in order to disentangle unspecific time effects from effects that are shared by the real and the sham training (and which may point towards other working mechanisms). However, designing an adequate control condition appears challenging, as some previous studies have found beneficial effects of control trainings on interpretation bias and/or symptomatology as well [23, 24, 26].

There are further limitations to our study that are worth noting. Concerning our assessment task for negative interpretation bias, the exclusion rate was comparably high due to participants who failed to unscramble a sufficient amount of grammatically correct sentences, which diminished our sample size. Additionally, it should be noted that our CBM-I intervention was a modified version of the negative interpretation bias assessment task. The rationale for using the SST not only for the intervention but also for bias assessment was that it should best measure the very target that the intervention addressed. However, results of the intervention group at post-assessment may be due to practice effects with the task. In future studies, negative interpretation bias should be assessed by an additional measure. Another critical point that may have affected results of the intervention group is the participants’ awareness of the study aims and potential training benefits. Participants in the intervention group may have expected improvements in their body image and body dissatisfaction due to training. Future studies should rely on concealing the study purpose at the beginning as well as blinding participants with respect to their group affiliation. Furthermore, the sample mainly consisted of female students, limiting the generalizability of our results to cohorts with clinical eating disorder symptoms and a male population as well. We also did not systematically assess whether participants had a history of, or were currently diagnosed with, an eating disorder, thus we cannot rule out that women with a (sub-)threshold eating disorder participated in our study.

When it comes to future research on improving body image and body dissatisfaction, it may be helpful to involve different modalities. Our CBM-I training focused on verbal processing only. However, participants showed an increase in body dissatisfaction after exposure to photographs of fashion models meeting the thin-ideal – which in turn involved visual processing. An attenuation of this effect may be more easily achievable by CBM that operates within the same modality and draws on visual stimuli. One study already yielded promising results in this domain: After CBM-I training, participants shifted their categorization of presented bodies as ‘*thin*’ or ‘*fat*’ towards larger bodies [27]. Some other studies drew on visual stimuli as well but used a different approach (instead of CBM-I): so-called *evaluative conditioning*. By selectively pairing photographs of participants’ bodies with positive social stimuli (i.e. smiling faces), significant reductions in participants’ shape and weight concerns as well as an increase in body satisfaction were achieved [33–35]. These results appear promising, although some adaptations of the procedure may be necessary for use in clinical practice [50, 51]. Finally, as individuals with heightened body image concerns and body dissatisfaction do not only suffer from a negative interpretation bias, another potential approach is attention bias modification, e.g. a training to direct attention away from negative appearance-related stimuli [52, 53].

## Conclusions

This study adds to the growing body of research on cognitive bias modification to improve eating disorder psychopathology. This newly developed intervention aimed to modify body image-related negative interpretation bias, which is common among individuals with an eating disorder and those that are at risk of developing one, e.g. individuals with elevated levels of body dissatisfaction. In its current form, however, the intervention does not demonstrate effective treatment of body dissatisfaction and requires further adjustment. Considering this, previously established paradigms appear more suitable for modifying cognitive biases and corresponding symptomatology.

## Data Availability

The datasets used and analyzed during the current study are available from the corresponding author upon reasonable request.

## Abbreviations

ANOVA: Analysis of variance
BISS: Body Image States Scale
BMI: Body Mass Index
BSQ: Body Shape Questionnaire
CBM: Cognitive bias modification
CBM-I: Cognitive bias modification addressing interpretation bias
SATAQ: Sociocultural Attitudes Towards Appearance Questionnaire
SST: Scrambled Sentences Task
WHO: World Health Organization
